# Deciphering the Molecular Nexus: An In-Depth Review of Mitochondrial Pathways and Their Role in Cell Death Crosstalk

**DOI:** 10.3390/cells13100863

**Published:** 2024-05-17

**Authors:** Yumeng Li, Madiha Rasheed, Jingkai Liu, Zixuan Chen, Yulin Deng

**Affiliations:** Beijing Laboratory for Separation and Analysis in Biomedicine and Pharmaceuticals, School of Medical Technology, Beijing Institute of Technology, Beijing 100081, China; 3120211424@bit.edu.cn (Y.L.); madiharasheed@bit.edu.cn (M.R.); 3220211514@bit.edu.cn (J.L.)

**Keywords:** mitochondria, apoptosis, pyroptosis, necroptosis

## Abstract

Cellular demise is a pivotal event in both developmental processes and disease states, with mitochondrial regulation playing an essential role. Traditionally, cell death was categorized into distinct types, considered to be linear and mutually exclusive pathways. However, the current understanding has evolved to recognize the complex and interconnected mechanisms of cell death, especially within apoptosis, pyroptosis, and necroptosis. Apoptosis, pyroptosis, and necroptosis are governed by intricate molecular pathways, with mitochondria acting as central decision-makers in steering cells towards either apoptosis or pyroptosis through various mediators. The choice between apoptosis and necroptosis is often determined by mitochondrial signaling and is orchestrated by specific proteins. The molecular dialogue and the regulatory influence of mitochondria within these cell death pathways are critical research areas. Comprehending the shared elements and the interplay between these death modalities is crucial for unraveling the complexities of cellular demise.

## 1. Introduction

Life is orchestrated by mortality, where cell death is an essential process in cellular life activities, alongside cell differentiation and proliferation, and serves to eliminate damaged cells, maintain physiological homeostasis, and respond to pathological stimuli [1]. According to the Nomenclature Committee on Cell Death (NCCD), cell death is categorized into accidental cell death (ACD) and regulated cell death (RCD) based on the mode of cell demise. This paper focused on RCD [2], a controlled signaling pathway crucial for organismal development, tissue renewal, and cellular morphology and function maintenance. RCD plays a significant role in various diseases, including immune and neurodegenerative diseases [3,4]. Various modalities of cell demise constitute RCD, with apoptosis being the foremost programmed cell death (PCD) elucidated. Historical traces of apoptosis research date back to the observations made by German scientist Karl Vogt in 1842. Notably, in 1965, John Foxton Ross Kerr and colleagues scrutinized cell death phenomena in rat liver tissues subjected to portal vein ligation, employing electron microscopy. Their investigation unveiled a highly organized form of cell death characterized by preserved lysosomes, gradual cellular shrinkage, condensed chromatin, detachment from neighboring tissue, and subsequent phagocytosis by immune cells. This meticulously orchestrated process, distinct from inflammatory necrosis, was initially termed contraction necrosis, later refined to apoptosis [5]. Contemporary investigative approaches to apoptosis entail techniques such as DNA fragmentation analysis, TUNEL (terminal deoxynucleotidyl transferase dUTP nick end labeling) staining, and the utilization of Western Blot analysis to assess the expression levels of related proteins, notably caspases. Pyroptosis, a distinct form of PCD discovered after apoptosis, was initially conceptualized by Brad Cookson et al. in 2005 [6]. Unlike apoptosis, pyroptosis exhibits distinctive morphological features, including increased membrane permeability, irregular cellular morphology, expansion of the endoplasmic reticulum, aberrant distribution of nuclear chromatin, followed by mitochondrial and nuclear swelling, lysosomal degradation, membrane rupture, and cytoplasmic release, often inciting inflammatory responses [7,8]. Moreover, pyroptosis is frequently associated with a robust inflammatory cascade, contrasting conventional cell death with the extracellular release of inflammatory cytokines such as IL-1β and IL-18 [9]. Recent studies have shown that methods enabling the elucidation of morphological alterations, membrane rupturing, detection of gasdermin (GSDM) cleavage via immunoblotting, and assessment of lactate dehydrogenase (LDH) activity serve as means to ascertain the occurrence of cellular pyroptosis [10]. In addition, investigations into the regulation of necrosis traced back to 1965 showed the secretion of lymphotoxin, cytotoxin, or tumor necrosis factor-α (TNF-α) by white blood cells to induce cell death, initially categorized as apoptosis. However, by 1988, it became evident that tumor necrosis factor (TNF) could also elicit necrosis in some cells. Subsequently, in 2000, the first genetic evidence of necrosis emerged: T cells stimulated with Fas ligand, in the presence of a caspase inhibitor, underwent cell death dependent on Fas-associated death domain protein (FADD)/receptor-interacting protein kinase 1 (RIPK1), exhibiting necrotic characteristics while retaining cytochrome C [11]. Shortly after, in 2005, Degterev et al. introduced the concept of necroptosis, an immunogenic form of cell death triggered by similar stimuli as apoptosis [12,13]. Nonetheless, necroptosis diverges from apoptosis in several aspects. Morphologically, necroptosis, akin to pyroptosis, entails organelle swelling and plasma membrane rupture. Furthermore, necroptosis is instigated by death receptors (e.g., FAS and tumor necrosis factor receptor 1 (TNFR1)) sensing signals within the cellular microenvironment [14].

The PCD death pathways are intricate and heterogeneous. Apoptosis, characterized by various triggering factors, manifests primarily through two distinct pathways, each regulated by unique factors. The two distinct mechanisms are characterized as the intrinsic apoptosis pathway, which is mediated by factors such as DNA damage and orchestrated by the caspase family, while the extrinsic apoptosis pathway is initiated by the activation of death receptors [2,15,16,17]. Necroptosis, an alternative form of cell death, ensues when apoptotic initiation fails and is mainly triggered by death receptors and toll-like receptors, with RIPK1/receptor-interacting protein kinase 3 (RIPK3)/mixed lineage kinase domain-like protein (MLKL) serving as the leading death execution molecules [18,19]. Pyroptosis primarily depends on the activation of the inflammasome to activate the caspase protein family, causing a variety of physiological responses [4].

Mitochondria are widely recognized as central to cellular metabolism, serving as a primary source of adenosine triphosphate (ATP) and reactive oxygen species (ROS). Dysfunction of these organelles is implicated in a spectrum of age-related diseases, including neurodegenerative conditions [20]. The main functions of mitochondria include ATP production, calcium ion storage, cellular signal transduction, and regulation of cell death. The structure of mitochondria is complex, comprising the outer membrane, inner membrane, matrix, and intermembrane space, each with specific biological functions. The mitochondrial outer membrane contains various protein channels, including the Bcl-2 protein family and mitochondrial import receptor subunit Tom20 [21]. The Bcl-2 protein family is divided into three classes: pro-apoptotic multi-domain proteins (Bax and Bak), pro-apoptotic BH3-only proteins (tBid, Bad, NOXA, PUMA, and Bim), and anti-apoptotic four-domain proteins (Bcl-2 and Bcl-xL) [22]. The folding of the mitochondrial inner membrane into cristae dramatically increases its surface area, which is the leading site of ATP synthesis. The selective permeability of the inner membrane is crucial for maintaining mitochondrial function [23]. The mitochondrial matrix and intermembrane space are rich in enzymes and signaling molecules, such as apoptosis-inducing factor (AIF), endonuclease G (Endo G), HtrA2, and cytochrome C [24,25]. These components, shapes, and related proteins of mitochondria play significant roles in different PCD pathways, and they differ among them. During apoptosis, mitochondria undergo substantial morphological changes, including loss of membrane potential, increased permeability, and volume reduction. These changes accompany the release of cytochrome C into the cytosol, thereby triggering apoptosis [26]. In pyroptosis, distinctive morphological features of mitochondria mainly include loss of membrane potential, swelling, and rupture of the outer membrane [27]. Necroptosis, unlike apoptosis and pyroptosis, does not typically involve morphological changes in mitochondria. However, mitochondrial dysfunction can occur, leading to disruption of the electron transport chain and a sharp decrease in cellular ATP levels. Thus, mitochondria also play a crucial role in the process of necroptosis [28].

Furthermore, the calcium ion storage capability of mitochondria and the generation of ROS play crucial roles in regulating PCD. During apoptosis, the influx of calcium ions can facilitate critical processes, such as the activation of calcium-dependent proteases such as calpain. These enzymes can promote cell death either directly or indirectly by activating members of the caspase family [29]. The production of ROS is also a significant factor in apoptosis; it can compromise mitochondrial membrane integrity, promote the release of cytochrome C, and thus lead to apoptosis [30]. In the process of pyroptosis, calcium ions modulate the activation of inflammasomes [31]. Similarly, ROS can enhance the assembly and function of inflammasomes, intensifying the inflammatory response of the cell [32]. In necroptosis, the increase in calcium ions and ROS can amplify the activation of RIPK3, thereby facilitating the translocation of the MLKL protein to the cell membrane and its disruption, leading to necroptotic cell death [33,34].

Recent studies have revealed that the regulation of mitochondria-associated proteins may be involved in several PCD pathways. For instance, intrinsic apoptosis is primarily caused by pore formation mediated by the Bcl-2 family proteins located on the mitochondrial membrane [35]. The role of the Bcl-2 protein family in pyroptosis is not entirely clear, but members of the Bcl-2 family, along with mitochondrial membrane proteins such as Tom20 and HK-II, can indirectly regulate the pyroptosis process. They do this by affecting mitochondrial stability and ROS production and by inhibiting the release of cytochrome C from mitochondria [36].During necroptosis, mitochondria primarily regulate the pathway through the release of specific proteases and pro-apoptotic factors that influence crucial necroptotic elements such as RIPK1, driving the cell toward death [37]. Thus, mitochondria and their associated proteins are closely linked to the regulation of PCD.

On the other hand, recent studies have revealed the inadequacy of attributing cell death solely to individual pathways within apoptosis, pyroptosis, and necroptosis, indicating instead a complex interplay of multiple effectors across these intricate PCD pathways [16,38]. Notably, caspase-8 has emerged as a significant molecule identified to serve as a conduit between various modes of cell death [38]. In addition to this, we believe that mitochondria act as decision-makers that can direct cells to different modes of death through various mediators. However, the intricate mechanisms underlying these processes necessitate further comprehensive exploration. In this review, we aim to elucidate the precise mechanisms by which mitochondria concurrently regulate multiple PCD pathways and focus on synthesizing the crucial cross-effector molecules modulated by mitochondria within both apoptosis/pyroptosis and apoptosis/necroptosis pathways.

## 2. Apoptosis: Programmed Cell Death

Apoptosis is the predominant form of regulated cell death, which ensues through a series of caspase cascades and is potentiated by factors including bacteria, viruses, hyperthermia, chemotherapeutic agents, and radiation [17]. It constitutes a genetically predetermined, active, and methodical mode of cellular demise, characterized by the induced activation of pertinent molecular mechanisms and genetic programming. Apoptosis primarily manifests through the formation of apoptotic vesicles triggered by either endogenous or exogenous stimuli, after which there is a decline in mitochondrial membrane potential, disruption of nuclear membrane integrity, and DNA degradation, culminating in apoptotic cell death. The process of apoptosis unfolds gradually and is typically delineated into stages such as early apoptosis, early-middle apoptosis, middle-late apoptosis, and late apoptosis [38].

### 2.1. The Extrinsic Apoptotic Pathway

The exogenous pathway is caused by extracellular perturbations within the cellular microenvironment, prompting the cell to release inflammatory metabolites and apoptotic extracellular vesicles (ApoEVs), among other factors. Concurrently, phosphatidylserine is exposed on the cell membrane surface of apoptotic cells, signaling phagocytes to engulf and clear them upon receipt of the signal [39,40]. The initiation of exogenous apoptosis entails the binding of specific ligands, such as TNFR1, tumor necrosis factor-related apoptosis-inducing ligand (TRAIL), or Fas ligand (Fas-L/CD95-L), to their respective receptors on the cell membrane [41]. This interaction leads to the assembly of the death-inducing signaling complex (DISC), comprising TNFR1-associated death domain protein TRADD, Fas-associated death domain protein FADD, FLICE inhibitory protein (FLIP), and procaspase-8/10. Within the DISC, procaspase-8 is catalyzed to active caspase-8, initiating a cascade of caspase activation reactions ultimately culminating in the activation of caspase-3/7, pivotal in orchestrating the apoptotic process and ensuing cell death [42]. Additionally, the activity of caspase-8 is not only negatively regulated by FLIP but also found to cleave BID to promote the mitochondria-associated endogenous apoptosis pathway [43,44]. Specifically, cleaved BID generates truncated tBID, which translocates to the mitochondrial membrane, inducing MOMP and consequently triggering intrinsic mitochondrial apoptosis [45,46,47].

### 2.2. The Mitochondrial Intrinsic Apoptotic Pathway

**Figure 1:**
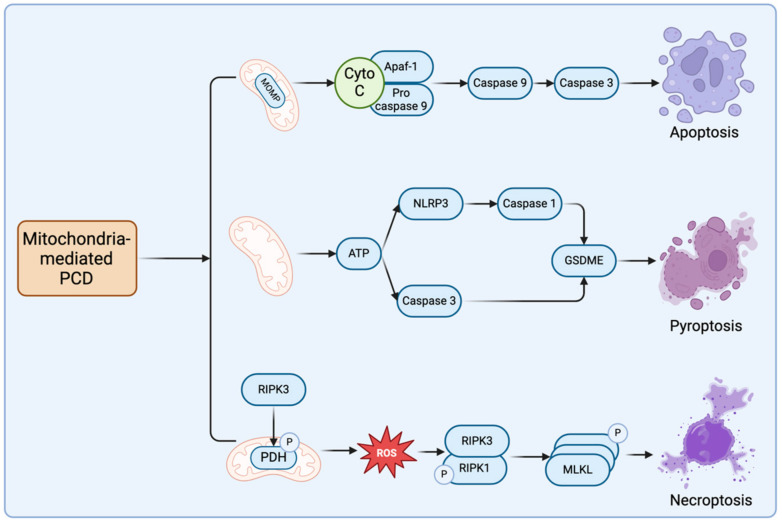
Components within each PCD pathway that are regulated by mitochondria. (The figure illustrates the key proteins and pathways regulated by mitochondria within the respective routes of apoptosis, pyroptosis, and necroptosis. In the apoptotic pathway, mitochondria mainly promote the formation of apoptotic bodies and the cascade amplification reaction of the caspase family by releasing cytochrome C, which eventually leads to apoptosis. In the pyroptosis pathway, mitochondria influence cell death by regulating ATP synthesis, which activates NLRP3 and caspase-3, ultimately leading to the cleavage of GSDME. In the necroptosis pathway, damaged mitochondria release ROS, which further promotes the binding and phosphorylation of RIPK1 and RIPK3, followed by the aggregation and phosphorylation of MLKL, resulting in necroptotic cell death).

The endogenous apoptotic pathway, also known as the mitochondrial pathway, is triggered by various intrinsic stresses such as chemotherapeutic agents, radiation, free radical damage, viral infection, and DNA damage. These stimuli disrupt intracellular homeostasis, leading to MOMP and the subsequent release of cytochrome C into the cytoplasm. cytochrome C binds to apoptotic protease activating factor 1 (Apaf-1), facilitating the recruitment of procaspase-9 to form the apoptosome complex. Procaspase-9 within the complex undergoes cleavage, generating active caspase-9, thereby initiating a cascade of caspase activation events [48]. Regulation of the endogenous apoptotic pathway is governed by the BCL-2 family of proteins localized in the mitochondrial membrane. This family is categorized into pro-apoptotic (e.g., BAX, BAK, PUMA, BAD, NOXA, BIK) and anti-apoptotic proteins (e.g., BCL-2, BCL-XL) [49,50]. BAX and BAK serve as principal effectors of endogenous apoptosis, undergoing conformational changes in response to cellular stress and oligomerizing at the mitochondrial membrane. Upon oligomerization, BAX and BAK form pores in the mitochondrial membrane, leading to MOMP. Furthermore, BH3-domain proteins such as BID and BIM facilitate BAX/BAK oligomerization on mitochondria and subsequent cytochrome C release [51]. In contrast, BCL-2/BCL-XL heterodimerizes with pro-apoptotic proteins through BCL-2 homology structural domain 3 (BH3) and hinders the binding of BAX/BAK complex or other pro-apoptotic proteins to the mitochondrial membrane to inhibit endogenous apoptosis [52], pointing out that expression levels and affinity of BCL-2 family proteins on mitochondria impact apoptosis to some extent [38]. The above content is presented in Figure 1.

## 3. Pyroptosis: Inflammatory Cell Death

Pyroptosis represents another modality of cell death, characterized by the activation of inflammatory vesicles resulting in cytoplasmic membrane disruption, the release of cellular contents, and the elicitation of an inflammatory response. In 2015, pyroptosis was acknowledged as a form of cell death mediated by the gasdermin family [53]. Comprising gasdermin A, gasdermin B, gasdermin C, gasdermin D (GSDMD), gasdermin E (GSDME), and PJVK [53,54], with GSDMD and GSDME being extensively investigated in cellular pyroptosis [54,55]. Gasdermins (GSDMs) share two conserved structural domains: the N-terminal pore-forming domain (PFD) and the C-terminal regulatory domain (RD). Upon cellular stimulation by endogenous or exogenous triggers, GSDMs undergo cleavage, releasing the PFDs, which then oligomerize to form pores on the cell membrane. This event leads to cellular enlargement, rupture, and the subsequent release of inflammatory factors, culminating in pyroptotic cell death [56]. Previous studies have suggested that GSDMs are cleaved by the caspase family, including caspase-1, at the aspartate site [57].However, recent findings reveal that granzyme A (GzmA) can also cleave GSDMs at non-aspartate sites to initiate pyroptosis. This discovery challenges the conventional notion that only cysteine-aspartic proteases can trigger pyroptosis [58]. Pyroptotic pathways are further categorized into classical and non-classical pathways. Here, we also address aspects of pyroptosis potentially regulated by mitochondria.

### 3.1. The Canonical Pyroptosis Pathway

Inflammasomes primarily orchestrate classical cell pyroptosis, serving as multimolecular complexes activated upon cellular infection to instigate an adaptive immune response. Various components such as inflammasomes typically include Toll-like receptors [59], NOD-like receptors, pattern recognition receptors (PRRs), apoptosis-associated speck-like protein containing a caspase recruitment domain (ASC), and downstream pro-caspase-1. Among them, the PRR recognizes danger-associated molecular patterns (DAMP) and pathogen-associated molecular patterns (PAMP) induced by pathogen invasion, triggering the release of pro-inflammatory factors and initiating downstream signaling pathways [60,61]. ASC, comprising a caspase recruitment domain (CARD) and a pyrin domain (PYD), directly recruits pro-caspase 1. Furthermore, certain CARD-containing PRRs can also directly recruit pro-caspase-1 upon recognition of PAMP or DAMP [9]. Notable inflammasome constituents include NOD-like receptors (NLRP1, NLRP3, NLRC4), AIM2, and PYD [60,62,63]. Following activation by inflammasomes, caspase-1 cleaves GSDMD at the Asp275 site, yielding a 31 kDa N-terminal fragment (N-GSDMD). This fragment translocates to the cell membrane, forming pores with an inner diameter of 10–14 nm, facilitating the release of mature interleukin-1β (IL-1β) and interleukin-18 (IL-18) into the extracellular milieu [64]. Subsequently, cells undergo swelling and rupture, culminating in pyroptotic cell death, postulating GSDMD as the principal executor of cell pyroptosis, and mediating the final stages of this inflammatory cell death pathway [8,65,66,67].

### 3.2. Alternative Pyroptosis Pathways

Non-classical pyroptosis pathways also regulate the core protein GSDMD during cell pyroptosis beyond caspase-1 activation and involve the interaction of caspase-4/5/11 with intracellular lipopolysaccharide (LPS) via their N-terminal caspase activation and recruitment domains (CARDs), thereby catalyzing the conversion of inactive pro-caspase-4/5/11 to active caspase-4/5/11 [8]. Active caspase-4/5/11 exhibits similar functionality to caspase-1, both cleaving GSDMD to generate the N-terminal fragment (NT-GSDMD) that localizes to the cell membrane, leading to pore formation and subsequent initiation of pyroptosis [67]. However, unlike the classical pyroptotic pathway, this non-classical pathway does not activate pro-interleukin-1β (pro-IL-1β) or pro-interleukin-18 (pro-IL-18), indicating that GSDMD solely mediates pyroptotic function in non-classical pyroptosis without amplifying the pyroptotic response [68].

Furthermore, GSDME is implicated in non-classical pyroptotic pathways. GSDME, abundantly expressed in various normal tissues, including the brain and heart, can be cleaved by caspase 3, an essential protein in apoptosis, thereby diverting cellular fate from apoptosis to pyroptosis. Recent studies have revealed that various chemotherapeutic agents or TNF-α induce caspase-3-mediated cleavage of GSDME at site 267DAMP270, generating NT-GSDME. Subsequently, NT-GSDME forms pores in the cell membrane, leading to cellular inflammation, rupture, and the release of inflammation-related factors and DAMP, triggering tumor cell pyroptosis [69]. Additionally, granzyme B has been identified as another enzyme capable of cleaving GSDME at the same site, thereby inducing cell pyroptosis [58]. Moreover, granzyme B exhibits substrates beyond GSDME, as it can cleave and activate caspase-3 at site D175, thereby influencing apoptosis or pyroptosis [70].

### 3.3. Mitochondrial Modulation of Pyroptosis

Few studies have demonstrated the direct regulatory role of mitochondria in pyroptosis. In apoptosis, caspase-3 is engaged in both the extrinsic and the intrinsic apoptotic pathways regulated by mitochondria, representing the terminal enzyme in the caspase cascade [71,72,73]. However, emerging evidence suggests that caspase-3, in conjunction with GSDME, plays a pivotal role in driving pyroptosis [21]. Mitochondria primarily function to generate ATP, which is essential for cellular respiration. Recent investigations have revealed that ATP can activate the NLRP3 inflammasome in classical pyroptotic pathways. Interestingly, even when NLRP3 activation is hindered, ATP retains its capacity to induce pyroptosis in macrophages through the caspase-3/GSDME pathway [74]. Photodynamic therapy, a modality for inducing cancer cell death, has been linked to GSDME-mediated pyroptosis. Specifically, this pathway triggers the activation of caspase-8 and caspase-3 by inhibiting pyruvate kinase M2 (PKM2), leading to the liberation of the N-terminus of GSDME [75]. The above is shown in Figure 1.

## 4. Necroptosis: A Caspase-Independent Cell Death

### 4.1. The Necroptotic Signaling Pathway

Necroptosis typically arises as a cellular response to severe chemical or physical stress; contrasting with apoptosis, it initiates an inflammatory reaction. It is characterized by cell swelling and rupture [76,77], similar to apoptosis, governed by specific pathways and associated proteins, leading to membrane integrity loss and the passive release of cellular contents [78]. The formation of necrosomes, comprising RIPK1 and RIPK3, occurs in the presence of FADD, culminating in the phosphorylation of MLKL. Subsequently, MLKL translocates to the plasma membrane along with tight junction proteins, initiating pore formation by interacting with phosphatidylinositol, facilitating inward ion flux and pore formation [79], ultimately resulting in cell swelling, rupture, and the release of cell DAMP. Necroptosis has been found not to involve reactive ROS production [80]. 

As necroptosis destroys cells to an irreversible extent, this cell death pathway is necessarily regulated in a variety of ways. Its regulation encompasses various mechanisms. Notably, pH sensitivity influences necroptosis, as the TNF-induced RIPK1-dependent pathway can be modulated by environmental pH. Intracellular pH reduction inhibits RIPK1 activity, thereby dampening necroptosis [81]. Additionally, cellular inhibitors of apoptosis protein 1 (cIAP1) and X-linked inhibitors of apoptosis protein (XIAP) regulate RIPK1/RIPK3 expression via ubiquitination. Downregulation of cIAP1 enhances TNFR1 production, rendering tumor cells more susceptible to RIPK1/RIPK3-induced necroptosis [82,83,84]. TRIF and interferon regulatory factors (DAI/ZBP) act as activators or inhibitors of necroptosis [38]. Cells lacking DAI exhibit resistance to influenza A virus-triggered necroptosis, whereas appropriate DAI concentrations protect autophagy-deficient cells from necroptosis [85,86]. Necroptosis is increasingly utilized in cancer therapy, with strategies targeting upstream necroptosis inhibition. Studies reveal that the mitochondrial protein Smac degrades cIAP1 and XIAP, preventing RIPK1 ubiquitination and promoting necroptosis continuation [87,88]. Nevertheless, a comprehensive understanding of necroptosis holds promise for novel therapeutic interventions in disease management [89].

### 4.2. Mitochondrial Involvement in Necroptosis

As shown in Figure 1, previously, it was believed that necroptosis exhibited minimal association with mitochondrial instability and reactive ROS generation and reported that despite mitochondrial fragmentation during necroptosis in L929 cells, depletion of dynamin-related protein 1 (DRP1) did not diminish necroptosis occurrence, implying that mitochondrial destabilization may not directly influence necroptosis progression [80,90,91]. However, recent inquiries have revealed that RIPK3 phosphorylates and activates the E3 subunit of the pyruvate dehydrogenase complex (PDH). Activated PDH, in turn, augments cellular aerobic respiration and mitochondrial ROS production, suggesting a plausible association between necroptosis and mitochondria [92]. Furthermore, substantial evidence indicates that RIPK3 activation promotes mitochondrial ROS generation, which subsequently intensifies RIPK1 phosphorylation, further augmenting necroptosis [93,94]. Despite these revelations, additional investigations are warranted to elucidate other necroptotic pathways and mechanisms influenced by mitochondria. We summarize the Morphologic Mechanisms of the three PCD pathways, Major Pathway Proteins, and Associated Cancers, as shown in Table 1.

## 5. Mitochondrial Regulation of Cell Death Pathway Transitions

### 5.1. Interplay between Mitochondrial Apoptosis and Pyroptosis

BAX and BAK serve as pivotal regulators in mitochondrial apoptosis by forming oligomers at the mitochondrial membrane in response to apoptosis-related stress, facilitating the release of cytochrome C from mitochondria, and subsequently initiating apoptosis [95]. During the oligomerization process of BAX and BAK, members of the BH3 family, such as BID and BAD, can modulate apoptosis by altering the conformation of BAX and BAK. However, under certain stress conditions, cells may undergo pyroptosis via the BAX/BAK pathway.

In melanoma cells, elevated iron levels induce an increase in ROS, leading to the oligomerization of the mitochondrial outer membrane protein Tom20. Subsequently, Tom20 recruits BAX to the mitochondrial membrane, triggering mitochondrial damage and activation of the BAX/cytochrome C/caspase-3/GSDME pathway, ultimately resulting in cell pyroptosis [21]. In contrast, inflammasome-mediated pyroptosis is initiated by activated caspase-1 cleaving GSDMD. Notably, even in the absence of GSDMD, cells can still undergo pyroptosis through a caspase-1-mediated pathway, accompanied by cytochrome C release and mitochondrial depolarization. This alternative mechanism involves the BID/apaf-1/caspase-9/caspase-3/GSDME pathway [96]. Additionally, prior investigations have identified an association between caspase-1 and BID, where BID can be cleaved by caspase-1 in vitro to generate an active truncated form, tBID [47].

Hexokinase (HK) serves as a pivotal enzyme in glycolysis, exerting a significant influence on apoptosis inhibition and metabolic promotion. Both isoforms I and II of HK possess mitochondrial targeting sequences. Research has indicated that in ATP-depleted renal epithelial cells, HK-II dissociates from the mitochondrial membrane, consequently leading to the release of apoptosis-associated factors, including AIF and cytochrome C from mitochondria, resulting in caspase-3 activation and apoptosis. It has been hypothesized that an antagonistic relationship exists between HK-II and BAX, with both proteins competitively binding to mitochondrial membrane-binding sites. Cells with elevated HK-II expression exhibit increased organelle-associated HK-II and reduced accumulation of BAX on mitochondria, mitigating mitochondrial outer membrane damage and apoptosis occurrence [36]. Recent investigations have revealed that cells undergo HK-II inhibition and mitochondria-associated pyroptosis under stress induced by tretinoin lactone (TPL), a compound isolated from traditional Chinese medicine with anticancer properties. Treatment of head and neck cancer cells with TPL led to decreased expression of HK-II and c-myc, resulting in translocation of the BAD/BAX complex to the mitochondrial membrane. This led to mitochondrial damage and cytochrome C release, activating caspase-3 and subsequent cleavage of GSDME, ultimately culminating in cell pyroptosis [97]. Consequently, the regulation of HK-II on the mitochondrial membrane can influence cell fate toward caspase-9/caspase-3-associated apoptosis or caspase-3/GSDME-associated pyroptosis, suggesting that modulation of cellular stress conditions may dictate the occurrence of different modes of cell death.

Additionally, it has been demonstrated that mitochondrial anti-apoptotic proteins, MCL-1 and BCL-xL from the BCL-2 family, are deactivated upon virus infection of human keratinocytes. However, instead of apoptosis, this deactivation triggers mitochondrial damage and caspase-3/GSDME-dependent pyroptosis [98]. BCL-2 also regulates cell pyroptosis through its interaction with GSDMD. Research by Chong-Shan Shi et al. revealed that the BH3 domain in GSDMD interacts with Bcl-2. Co-expression of caspase-1 and Bcl-2 in HEK293T cells resulted in active caspase-1 cleaving GSDMD at the D87 site rather than the classical D275 site in the pyroptosis pathway. This cleavage pattern reduces GSDMD-NT, which has pore-forming capabilities, while increasing cleavage at D87, thereby inactivating the pyroptosis execution program [99]. 

**Figure 2:**
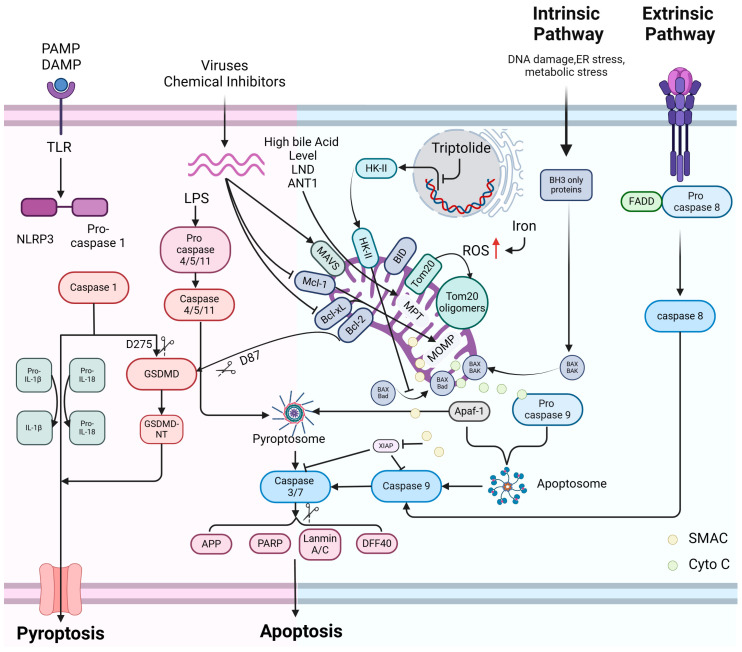
The intersection of apoptosis and pyroptosis is regulated by mitochondria. a. Exogenous apoptosis pathway: ligand/Death receptor/FADD/Pro-caspase-8 binds at the plasma membrane and mediates apoptosis through the downstream Caspase 8/CASP3/PARP pathway; b. Endogenous apoptosis pathway: under the stimulation of endogenous factors such as DNA damage/ER stress/metabolic stress, mitochondria undergo MOMP through the mitochondrial pro-apoptotic proteins BAX/BAK/BAD, and then apoptosis is mediated through the downstream cytochrome C/apaf-1/Caspase 9/Caspase 3/PARP pathway; c. Classical pyroptosis pathway: upon PAMP/DAMP stimulation, NLRP3/ASC/Pro-caspase-1 forms pyroptosis vesicles, which mediate pyroptosis via active Caspase 1 to activate Pro-IL-1/Pro-IL-18 and cleave GSDMD; d. Non-classical pyroptosis pathway: the presence of intracellular LPS contributes to the occurrence of Caspase 4/11/GSDMD-associated pyroptosis; e. Intersecting pathways of apoptosis/pyroptosis regulated by mitochondria: elevated intracellular Iron causes mitochondria to mediate the onset of pyroptosis via the elevated ROS/Tom20 oligomerization/BAX/MOMP/cytochrome C release/apaf-1/Caspase 9/Caspase 3/GSDME pathways. Caspase 1 in pyroptosis vesicles can mediate pyroptosis via the mitochondrial pro-apoptotic protein t BID/apaf-1/Caspase 9/Caspase 3/GSDME pathway. High expression of HK-II inhibits both the pathway of cells toward cytochrome C/apaf-1/Caspase 9/Caspase 3/PARP-associated pyroptosis and the pathway of cells toward the cytochrome C/apaf-1/Caspase 9/Caspase 3/GSDME-associated pyroptosis pathway, and Triptolide inhibits the expression of HK-II. Inactivation of mitochondrial anti-apoptotic proteins such as Mcl-1 and Bcl-xL instead led to mitochondrial-mediated pyroptosis via the MOMP/cytochrome C release/apaf-1/Caspase 9/Caspase 3/GSDME pathway. Bcl-2 can inhibit pyroptosis by interacting with Caspase 1, which enhances GSDMD cleavage at the D87 site. In the presence of high bile acid levels, LND, and ANT1, mitochondria mediate pyroptosis via MPT/cytochrome C/Apaf-1/Caspase 4/Caspase 3/GSMDE.

It was found that at elevated levels of bile acids, mitochondria experience a permeability transition (MPT), leading to the release of cytochrome C and other substances. This event triggers the formation of a protein complex called pyroptosis vesicles in the cytoplasm, consisting of Apaf-1 and caspase-4, which subsequently induces the cleavage of GSDME by caspase-3, initiating pyroptosis [100]. Prior investigations have consistently implicated Apaf-1 in the mitochondria-associated apoptotic pathway, where it, along with caspase-9, participates in the assembly of apoptotic vesicles without involvement in cellular pyroptosis initiation [101]. Conversely, caspase-4 is activated solely by cytoplasmic lipopolysaccharides (LPS), subsequently activating GSDMD and initiating cell pyroptosis [102]. However, a study by Wanfeng Xu et al. demonstrated that bile acids, calcium overload, and specific activators of adenine nucleotide transporter proteins induce MPT in mitochondria, leading to the activation of Apaf-1. This activated Apaf-1 then forms pyroptosis vesicles with pro-caspase-4/11, promoting the conversion of pro-caspase-4/11 into their active forms. Consequently, caspase-3 is cleaved, and GSDME is activated, resulting in cell pyroptosis. These findings underscore the multifaceted role of mitochondria in regulating cell death processes, including apoptosis, necrosis, and pyroptosis [100]. Hence, it is hypothesized that under different stress conditions, Apaf-1 may interact with caspase-4 to induce cell pyroptosis or bind to caspase-9 to trigger apoptosis. Hence, altering the stress conditions affecting mitochondria may serve as a crucial determinant for cells to undergo mitochondria-associated pyroptosis or apoptosis, as shown in Figure 2.

### 5.2. Crosstalk between Mitochondrial Apoptosis and Necroptosis

Among the mitochondria-related apoptotic pathways described above, MOMP is instigated when cells undergo DNA damage, encounter excessive metabolic stress, or experience endoplasmic reticulum stress [103]. This leads to the release of factors, including mtDNA and cytochrome C, from mitochondria into the cytoplasm [104]. Some of these factors play a regulatory role in necroptosis; for instance, critical aspects of the necroptosis pathway, namely RIPK1/RIPK3/MLKL, are under the regulation of mitochondria [105,106]. Positioned within the mitochondrial membrane, PUMA, a member of the pro-apoptotic Bcl-2 family, not only mediates mitochondria-associated apoptotic pathways but also contributes to necroptosis. Upon stimulation by TNF-α, PUMA facilitates the release of mitochondrial DNA into the cytoplasm, thereby promoting the activation of DAI/Zbp1 and STING, which subsequently enhances the phosphorylation of RIPK3 and downstream MLKL, culminating in necroptosis. Interestingly, phosphorylated RIPK3 and MLKL further amplify PUMA expression, establishing a positive feedback loop [107]. Moreover, in a study by Jin Young Baik et al., it was observed that glucose deprivation-induced necroptosis in rat tumor cells led to elevated expression of Zbp1. Additionally, increased levels of NOXA, a member of the BCL-2 protein family, activated MLKL, ultimately triggering necroptosis [108]. 

**Figure 3:**
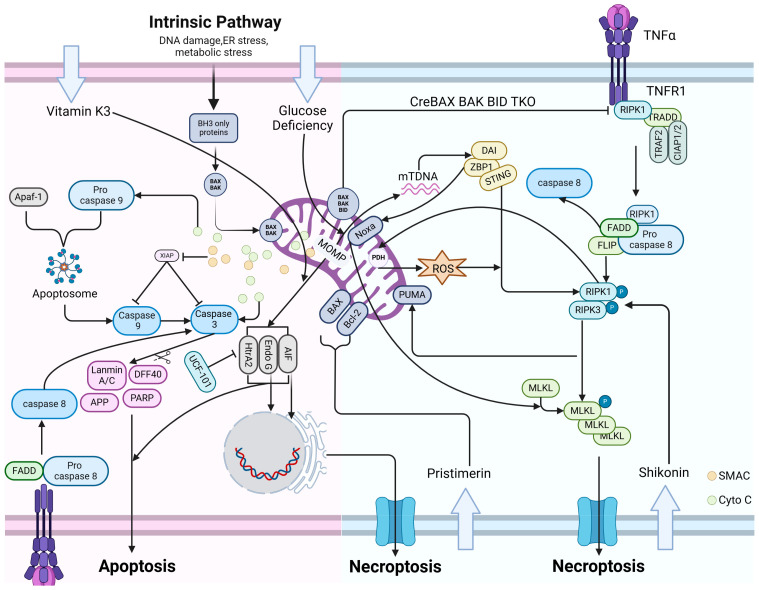
Intersection of apoptosis and necroptosis regulated by mitochondria. a. Endogenous apoptosis pathway: When cells are stimulated by endogenous factors such as DNA damage/ER stress/metabolic stress, the mitochondrial pro-apoptotic proteins BAX/BAK/BAD lead to the occurrence of MOMP, and then apoptosis is mediated by downstream cytochrome C/apaf-1/Caspase 9/Caspase 3/PARP. During the process of apoptosis, mitochondria release proteases or pro-apoptotic factors such as AIF, Endo G, HtrA2 to enhance apoptosis. b. Exogenous apoptotic pathway: ligand/Death receptor/FADD/Pro-caspase-8 binds at the plasma membrane and mediates apoptosis through the downstream CASP8/CASP3/PARP pathway. c. Necroptosis pathway: TNF-a binds to TNFR1/RIPK1/TRADD/TRAF2/CIAP1/2 at the plasma membrane, which activates the formation of necrosomes (RIPK1/FADD/Pro-Caspase-8/FILP) and further promotes downstream aggregation and phosphorylation of RIPK1/RIPK3 and MLKL. This ultimately contributes to necroptosis. d. Intersecting pathways of apoptosis/necroptosis regulated by mitochondria: stimulated by TNF-a, the pro-apoptotic protein PUMA on the mitochondrial membrane can also cause the mitochondria to release mtDNA, leading to necroptosis via the DAI/Zbp1/STING/P-RIPK3/P-MLKL pathway. P-RIPK3/P-MLKL can, in turn, further enhance PUMA expression. Elevated expression of Zbp1 can also trigger necroptosis via the NOXA/MLKL pathway. HtrA2 can inhibit apoptosis via Bcl-2. On the other hand, in DSS-induced colitis, HtrA2 can also lead to necroptosis via RIPK1. Under conditions of vitamin K3 exposure, mitochondria-released AIF and Endo G translocated to the nucleus, and the cells exhibited a necroptosis morphology. Under stress conditions such as Pristimerin, cells can upregulate the BAX/Bcl-2 ratio, produce excess ROS, and translocate AIF to the nucleus, culminating in chromatin cleavage. Cells undergo necroptosis. Knockdown of the pro-apoptotic proteins BAX/BAK/BID can lead to necroptosis by increasing the expression of RIPK1.

The mitochondrial apoptosis pathway releases various proteases or pro-apoptotic factors, including AIF, Endo G, and HtrA2, in addition to mtDNA and cytochrome C, as shown in Figure 3. These factors operate downstream of the caspase cascade in the cellular mitochondria-associated apoptotic pathway [24]. Among them, HtrA2/Omi, an oligomeric serine protease located in the mitochondrial membrane intermembrane space, has been implicated in neurodegenerative disorders such as Parkinson’s and Alzheimer’s diseases, as well as in maintaining mitochondrial homeostasis [109]. Several studies have also indicated that HtrA2 can serve as an anti-apoptotic agent by impeding the accumulation of Bcl-2 on the outer mitochondrial membrane [110]. Recent investigations have revealed that these factors, or proteases, not only facilitate apoptosis but also exert a significant role in necroptosis. For instance, in dextrose sodium sulfate (DSS)-induced colitis, HtrA2 translocates from the mitochondria to the cytoplasm and interacts with RIPK1, a pivotal component of the necroptosis pathway, thereby inducing necroptosis. Silencing or inhibiting HtrA2 expression substantially mitigated necroptosis occurrence in HT-29 and L929 cells [37]. Masayuki Fukui et al. demonstrated that the mitochondria of immortalized mouse hippocampal neuronal cells (HT22) exposed to menaquinone (vitamin K(3)) become dysfunctional and release two ATP-independent mitochondrial nucleases, AIF and Endo G. Remarkably, the morphological characteristics of the cells at the time of cell death resemble necroptosis rather than apoptosis [111]. Further investigations have unveiled that Pristimerin, a natural compound, exhibits anti-tumor properties by inducing a series of alterations in glioma cells, including upregulation of the BAX/Bcl-2 ratio, mitochondrial depolarization, and the generation of excessive ROS. Subsequently, AIF translocates from the nucleus to the mitochondria, triggering AIF-dependent necroptosis in glioma cells [112]. Moreover, under certain stress conditions leading to necroptosis, mitochondrial dysfunction occurs, and AIF migrates to the nucleus. This notion is supported by the study of Ye Ding et al., which explored the role of MLKL in the necroptosis of glioma cells and found that MLKL induces necroptosis by promoting chromatin disassembly. The principal mechanism involves MLKL causing AIF translocation to the nucleus following mitochondrial depolarization, leading to chromatin cleavage and subsequent necroptosis [113]. 

In the endogenous apoptotic pathway, BAX, BAK, and BID influence the expression level of RIPK1 [114]. Knockdown of BAX, BAK, and BID in mice was found to elevate RIPK1 kinase expression, ultimately leading to necroptosis in bone marrow hematopoietic stem cells, with BID playing a predominant role. Additionally, the study identified BID’s inhibitory mechanism on necroptosis, wherein it degraded RIPK1 by regulating caspase-8, thereby reducing necroptosis occurrence [115].

## 6. Conclusions

This review seeks to elucidate cell fate modulation through mitochondrial regulation of PCD. Apart from summarizing established pathways involved in apoptosis, pyroptosis, and necroptosis, we underscore the role of mitochondria in these PCD modalities. There is a burgeoning body of evidence indicating an interplay between different modes of cell death, with mitochondria playing a crucial role in this crosstalk. Mitochondria regulate the transition from apoptosis to pyroptosis under corresponding stimulatory conditions by modulating the expression of proteins such as BAX, BAK, HK-II, MCL-1, and BCL-xL at the membrane. This regulation leads the cell toward the cytochrome C/Caspase-3/GSDME pathway, facilitating the transition from apoptosis to pyroptosis. We also propose that apaf-1, a pivotal protein in the apoptosome, may form pyroptosis vesicles with caspase-4 under certain conditions, inducing pyroptosis while also binding to caspase-9 under different stresses, potentially representing another significant intersecting pathway between apoptosis and pyroptosis.

**Figure 4:**
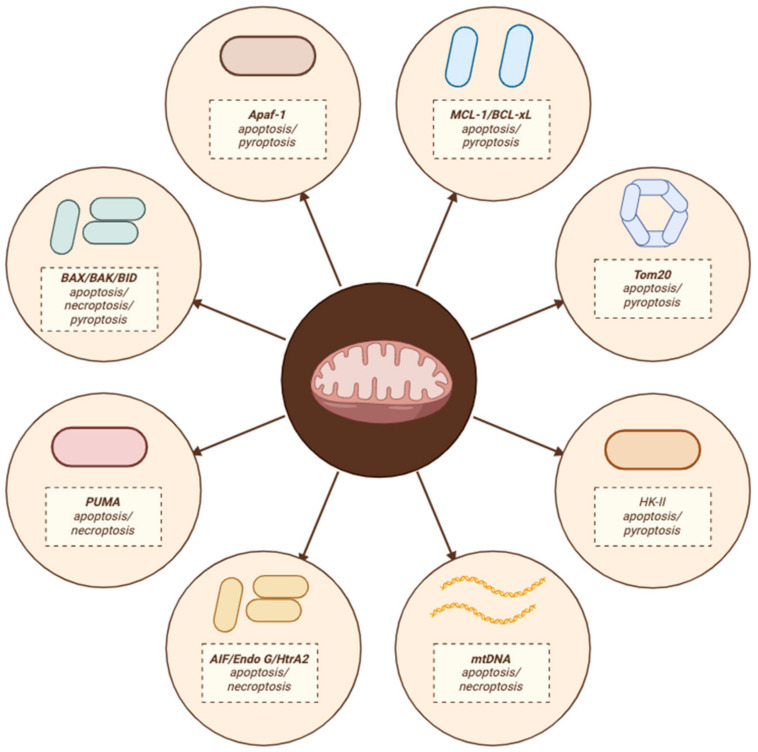
Intersection molecules of the mitochondrially regulated PCD pathway.

On the other hand, cells encountering stress cause mitochondrial outer membrane permeabilization (MOMP), and the release of proteases or factors from mitochondria, such as mtDNA, cytochrome C, AIF, Endo G, and HtrA2, can redirect cell fate from apoptosis to necroptosis. Additionally, regulation of the expression levels of BAX, BAK, and BID can also serve as a critical intersection between necroptosis and apoptosis. Nonetheless, these molecular mechanisms necessitate further elucidation. The intersection molecules of the mitochondrial regulatory PCD pathway are shown in Figure 4. However, future research should prioritize identifying novel cell death patterns regulated by mitochondria and elucidating their intersections under various stress conditions.

Additionally, it is crucial to develop and assess mitochondrial PCD-based crossover therapies in upcoming clinical trials. These efforts will not only deepen our understanding of cell death mechanisms but also pave the way for innovative therapeutic strategies targeting mitochondrial regulation of cell death. Such interventions hold promise for addressing a wide range of diseases, including cancer, neurodegenerative disorders, and inflammatory conditions, ultimately improving patient outcomes and quality of life. 

## Figures and Tables

**Table 1:** Morphologic Mechanisms, Major Pathway Proteins, Occurring Organs, and Associated Cancers of Apoptosis, Pyroptosis, and Necroptosis.

	Apoptosis	Pyroptosis	Necroptosis
Morphological characteristics	Blistering of cell membranes, reduction of cell volume, condensation of nuclei, chromatin condensation, loss of mitochondrial membrane potential, and cytoskeletal disintegration.	Swelling of cells, shrinkage of mitochondria, increased density of the mitochondrial membrane, and the formation of pores in the cell membrane.	Cell swelling, pore formation in the cell membrane, rupture of the plasma membrane and chromatin condensation.
Mechanism	Exogenous pathway: receptor-ligand binding, DISC formation, caspase-8/caspase-3 activation; Endogenous pathway: cellular stress, cytochrome c release, apoptotic vesicle formation.	Inflammatory vesicle formation, gasdermin cleavage by caspase-1/11, and the release of IL-1β and IL-18.	Predominance of caspase- family-independent RIPK1/RIPK3, phosphorylation of MLKL, and assembly of necrosomes.
Major pathway protein	caspase-8, caspase-3/7, PARP	caspase-1/11, GSDMD	RIPK1, RIPK3, MLKL
Associated cancer	Breast Cancer Lung Cancer Kidney Cancer Central Nervous System Disease Melanoma Lymphoma Leukemia Ovarian Cancer Uterine Cancer	Melanoma Breast Cancer Colorectal Cancer Stomach Cancer Hepatocellular carcinoma Lung Cancer Cervical Cancer Leukemia	Breast Cancer Colorectal Cancer Melanoma Cellular carcinoma Leukemia Glioblastoma Lung Cancer Pancreatic Cancer Stomach Cancer Ovarian Cancer Squamous cell carcinoma of the cervix
